# Parallel expression profiling of hepatic and serum microRNA-122 associated with clinical features and treatment responses in chronic hepatitis C patients

**DOI:** 10.1038/srep21510

**Published:** 2016-02-22

**Authors:** Azeem Mehmood Butt, Arsalan Jamil Raja, Shafiqa Siddique, Jahangir Sarwar Khan, Muhammad Shahid, Ghias-Un-Nabi Tayyab, Zahid Minhas, Muhammad Umar, Muhammad Idrees, Yigang Tong

**Affiliations:** 1 Molecular Virology Laboratory, Centre of Excellence in Molecular Biology (CEMB), University of the Punjab, Lahore, Pakistan; 2 Medicine Unit-I, Department of Medicine, Lahore General Hospital (LGH), Lahore, Pakistan; 3 Surgical Unit-I, Department of Surgery, Holy Family Hospital, Rawalpindi, Pakistan; 4 Medicine Unit-I, Department of Medicine, Centre for Liver and Digestive Diseases (CLD), Holy Family Hospital, Rawalpindi, Pakistan; 5 State Key Laboratory of Pathogen and Biosecurity, Beijing Institute of Microbiology and Epidemiology, Beijing, People’s Republic of China

## Abstract

MicroRNAs (miRNAs) are small, non-coding RNAs that regulate a variety of biological processes. Recently, human liver-specific miRNA miR-122 has been reported to facilitate hepatitis C virus (HCV) replication in liver cells. HCV is one of the leading causes of liver diseases worldwide. In Pakistan, the estimated prevalence is up to 10%. Here, we report hepatic and serum miR-122 expression profiling from paired liver and serum samples from treatment-naive chronic hepatitis C (CHC) patients and controls. We aimed to elucidate the biomarker potential of serum miR-122 for monitoring disease progression and predicting end treatment response (ETR). Hepatic miR-122 levels were significantly down-regulated in CHC patients. A significant inverse correlation was observed between hepatic and serum miR-122 levels, indicating that serum miR-122 levels reflect HCV-associated disease progression. Both hepatic and serum miR-122 were significantly correlated (*P* < 0.05) with several clinicopathological features of CHC. Receiver operator curve analysis showed that serum miR-122 had superior discriminatory ability even in patients with normal alanine transaminase levels. Multivariate logistic regression analysis highlighted pre-treatment serum miR-122 levels as independent predictors of ETR. In conclusion, serum miR-122 holds the potential to serve as a promising biomarker of disease progression and ETR in CHC patients.

Hepatitis C virus (HCV) is a small, enveloped, positive-sense single stranded RNA virus belonging to the family *Flaviviridae*. The viral genome is 9.6 kb in length with flanking 5′ untranslated region (UTR) and 3′ UTR regions, and consists of a single open reading frame (ORF) that codes for a polypeptide of approximately 3000 amino acids. This polypeptide is cleaved by host and viral proteases into viral structural and non-structural proteins^1^,^2^,^3^. On the basis of whole genome sequence variations, HCV is further divided into seven major genotypes and 67 subtypes^2^,^3^. HCV is one of the leading causes of chronic liver disease worldwide with an estimated prevalence of 170–200 million patients. The disease severity varies among patients and the persistent viral infection often leads to the development of serious complications such as cirrhosis and hepatocellular carcinoma^4^. Pakistan carries one of the highest burdens of HCV infection where the prevalence of HCV has been estimated to be 10%, and is dominated by HCV of genotype 3^5^,^6^.

Despite several advancements in the control of viral replication and the treatment of HCV patients on a global scale, the development of much-needed specific antiviral therapies and an effective vaccine remains limited^7^. To date, interferon has been considered as the first line of defence against HCV infections. Recent advancements have been made in the field of direct antiviral agents (DAA’s) against HCV, particularly of genotype 1. However, the standard treatment for CHC patients in many parts of the world, including Pakistan, remains pegylated-interferon plus ribavirin (PEG-IFN/RBV), irrespective of the fact that 50–60% of the patients fail to achieve a sustained virological responder (SVR) status and become non-responders (NR) or relapsers (RR)^8^. Therefore, prediction of the end treatment response (ETR) before the initiation of treatment is a crucial factor that could help in designing better therapeutic strategies and to overcome the undesired socio-economic burden and adverse side effects of PEG-IFN/RBV treatment. In this regard, several host and viral factors have been proposed as pre-treatment predictive biomarkers, the most notable of which is interleukin 28B (IL-28B). However, the predictive power of all these factors still remains variable globally, including within the Pakistani population^9^,^10^,^11^,^12^.

MicroRNAs (miRNAs) are small noncoding RNA molecules (18–24 nucleotides) that have been reported to play important roles in the regulation of several biological processes such as cell proliferation, differentiation, and apoptosis^13^,^14^. This is attributed to the ability of miRNAs to bind to specific sites at the 3′ UTR of target mRNAs, which in turn leads to translational repression and/or mRNA degradation^15^. Apart from the miRNA-mRNA pairing within an organism, it has also been observed that host miRNAs can also bind to mRNAs of invading pathogens such as viruses^16^,^17^. The most notable example in this regard is the complementary base pairing of the liver-specific human miRNA, miRNA-122 to the 5′ UTR of HCV genomic RNA, which is reported to facilitate viral replication within liver cells^18^. In addition to this, miR-122 is also reportedly involved in the regulation of lipid metabolism^19^, circadian gene expression^20^ and hepatic insulin resistance^21^. Therefore, miR-122 has recently gained much attention from the scientific community to better understand its potential as a therapeutic target, as well as a diagnostic marker in the case of HCV infections.

In addition to the tissue-specific origin and expression of several miRNAs, it has recently been shown that miRNAs can also be detected in various body fluids such as serum, plasma and urine^22^. In these samples, they exhibit stable expression patterns and are resistant to multiple freeze-thaw cycles and other conditions that can degrade other types of RNA^22^. Similar to tissue specimens, the pattern of circulating miRNAs has been found to be different in diseased patients and healthy controls for several types of cancers and other diseases^23^,^24^. This has led to the suggestion that circulating miRNAs could serve as useful non-invasive biomarkers of disease progression and prognosis. The biomarker potential of miR-122 in CHC infections, the majority of which were associated with HCV genotypes 1 and 2, have been evaluated previously resulting in both similar and conflicting findings^25^,^26^,^27^,^28^,^29^,^30^,^31^. However, no large scale analysis of miR-122 expression profiling in paired hepatic and serum samples has yet been reported in CHC patients, particularly not in the Pakistani population. Therefore, considering the significance of miR-122 in HCV infections and the overall prevalence of HCV in the population, a large-scale analysis of the biomarker potential of miR-122 is therefore warranted.

In the present study, we have assessed the potential associations of miR-122 expression levels with the clinicopathological features and outcomes of PEG-INF/RBV therapy in HCV genotype 3 CHC patients.

## Results

### Study participants

The baseline clinicopathological characteristics of the study subjects are summarized in Table 1. A total of 183 subjects participated in the study, of which 123 were treatment-naive CHC patients of HCV genotype 3 and 60 were healthy controls. The mean ages of the control and CHC patients groups were 39.2 ± 12.9 and 32.7 ± 9.9 respectively. No significant difference was observed in age and gender between different groups of patients and controls (*P* > 0.05). In the case of CHC patients, 61 were males and 62 females. There were equal number of males (n = 30) and females (n = 30) in the control group. The mean log baseline HCV viral load levels were 5.3 ± 0.9. All CHC patients were monitored for the duration of the PEG-INF/RBV therapy. According to the ETR, of the 123 CHC patients, 70 responded to therapy and were deemed as cleared of viral infection (indicated by SVR), whereas 53 failed to clear the viral infection (deemed NR or RR). Further classification of non-responders showed that among the non-responders (NR+RR) group (n = 53), 20 patients initially responded to the therapy but were found to be positive for HCV infection (indicated by RR in Table 1) at the end of therapeutic or observational phase (Table 1).

### Hepatic miR-122 levels are down-regulated in CHC patients

**Figure 1:**
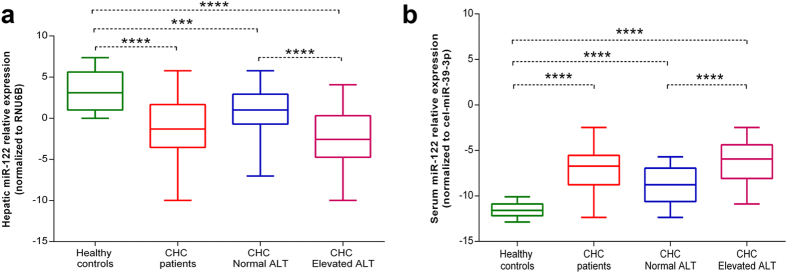
Relative expression levels of microRNA-122. The expression levels of hepatic (**a**) and serum (**b**) miR-122 in chronic hepatitis C patients with normal and elevated alanine transaminase (ALT) levels were compared with those of healthy controls. Boxes represent range, median and quartiles of the normalized miR-122 expression (∆Cq) levels. Asterisks indicate level of significant difference between analysed groups and are as follows: ****P < 0.0001, ***P < 0.001, **P < 0.01 and *P < 0.05.

In order to investigate if the expression of miR-122 is altered in the liver tissues of CHC patients, miR-122 levels in control and CHC liver tissue samples were measured via real-time quantitative PCR (RT-qPCR). It was found that miR-122 levels were significantly down-regulated in CHC liver tissues compared with controls (*P* = 2.15 × 10^−9^) (Fig. 1a). As the majority of CHC patients have stable and slightly elevated alanine transaminase (ALT) levels, we next determined whether miR-122 levels have any relationship with ALT levels. CHC patients were divided into two groups according to ALT levels; normal ALT group (ALT ≤ 40 IU/L; n = 43) and elevated ALT group (ALT > 40 IU/L; n = 80). Hepatic miR-122 levels were significantly different between the normal (*P* = 1.65 × 10^−4^) and elevated (*P* = 8.77 × 10^−11^) ALT groups of CHC patients compared with those of controls. Moreover, both groups of CHC patients also showed a significant difference in the expression levels of miR-122 between each other (*P* = 9.03 × 10^−7^), indicating that miR-122 expression is a more sensitive indicator of HCV infection than the routinely measured ALT levels (Fig. 1a).

### Serum miR-122 levels are up-regulated in CHC patients

To investigate if the levels of circulating miR-122 were also altered in CHC patients, concentrations of miR-122 were measured in sera from patients with CHC and controls. In contrast to hepatic miR-122, serum miR-122 levels were found to be significantly up-regulated in CHC patients compared with those of controls (*P* = 1.52 × 10^−14^). A similar trend of up-regulation with was observed when the serum miR-122 levels of the normal ALT (*P* = 5.21 × 10^−8^) and elevated ALT (*P* = 1.62 × 10^−15^) CHC patient groups were compared with those of controls (Fig. 1b).

### Hepatic and serum miR-122 levels correlate with each other in CHC patients

A significant inverse correlation (*r* = −0.395, *P* = 3.6 × 10^−5^) was observed between the levels of hepatic and serum miR-122 in CHC patients. This is in agreement with the above reported down- and up-regulation of miR-122 levels in matched liver tissues and serum samples of CHC patients, respectively.

### Hepatic miR-122 levels correlate with necroinflammation, fibrosis, and clinicopathological features of CHC patients

**Figure 2:**
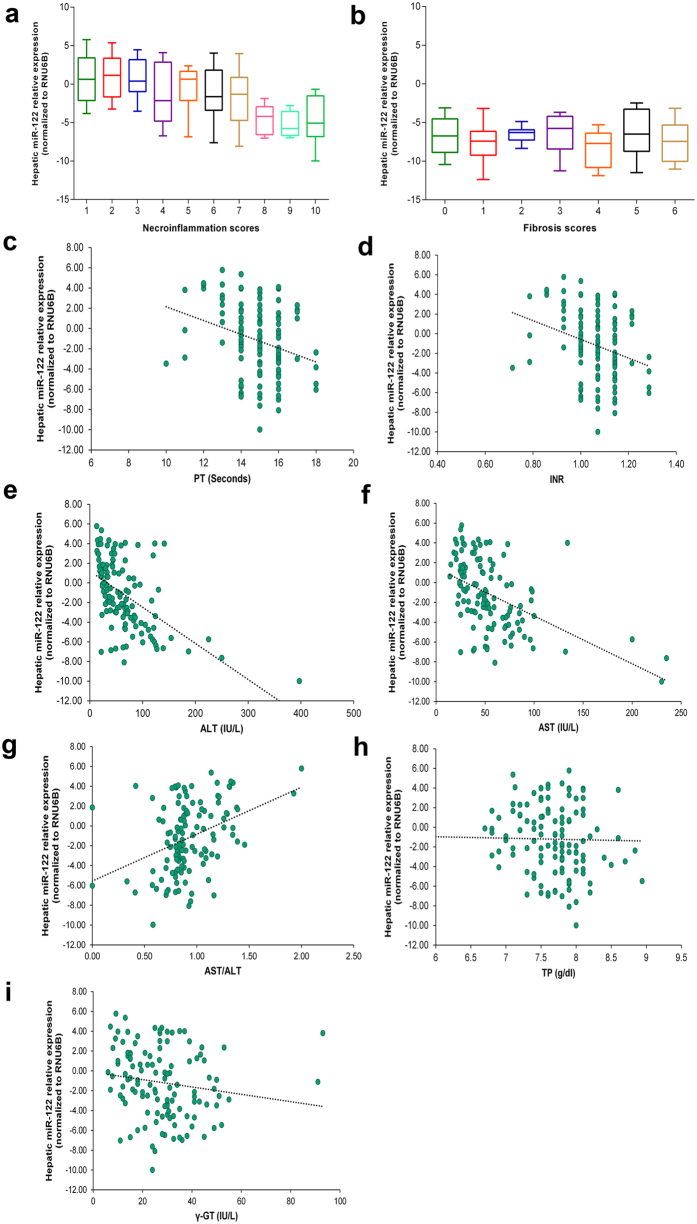
Correlation analysis of hepatic miR-122. The expression levels of hepatic miR-122 were significantly correlated with the following clinicopathological features in chronic hepatitis C patients: (**a**) Necroinflammatory scores. (**b**) Fibrosis scores. (**c**) Prothrombin time (PT). (**d**) International normalized ratio (INR). (**e**) Alanine transaminase (ALT). (**f**) Aspartate transaminase (AST). (**g**) AST/ALT ratio. (**h**) Total proteins (TP), and (**i**) Gamma-glutamyl transferase (γ-GT).

To evaluate the potential of hepatic miRNA-122 levels to indicate ongoing CHC-associated liver damage and disease severity, we correlated miR-122 levels with the histopathology index (HAI) and liver function parameters. Hepatic miR-122 levels were significantly correlated with the necroinflammation grading (*r* = −0.493, *P* = 6.79 × 10^−9^, 95% CI: −0.616 to −0.346) and fibrosis staging (*r* = −0.215, *P* = 0.015, 95% CI: −0.378 to −0.0398) scores (Fig. 2a,b). Among the analysed biochemical parameters, hepatic miR-122 levels showed significant correlations with prothrombin time (PT) (*r* = −0.282, *P* = 0.002, 95% CI: −0.438 to −0.111), international normalized ratio (INR) (*r* = −0.272, *P* = 0.001, 95% CI: −0.438 to −0.112), ALT (*r* = −0.559, *P* = 1.8 × 10^−11^, 95% CI: −0.670 to −0.424), aspartate transaminase (AST) (*r* = −0.518, *P* = 1.204 × 10^−9^, 95% CI: −0.637 to −0.374), AST/ALT ratio (*r* = 0.372, *P* = 2.3 × 10^−5^, 95% CI: 0.208 to 0.515), total proteins (TP) (*r* = −0.185, *P* = 0.040, 95% CI: −0.351 to −0.00840), and gamma-glutamyl transferase (γ-GT) (*r* = −0.256, *P* = 5.0 × 10^−3^, 95% CI: −0.416 to −0.0812) (Fig. 2c–i).

### Serum miR-122 levels correlate with necroinflammation and clinicopathological features but not with fibrosis in CHC patients

**Figure 3:**
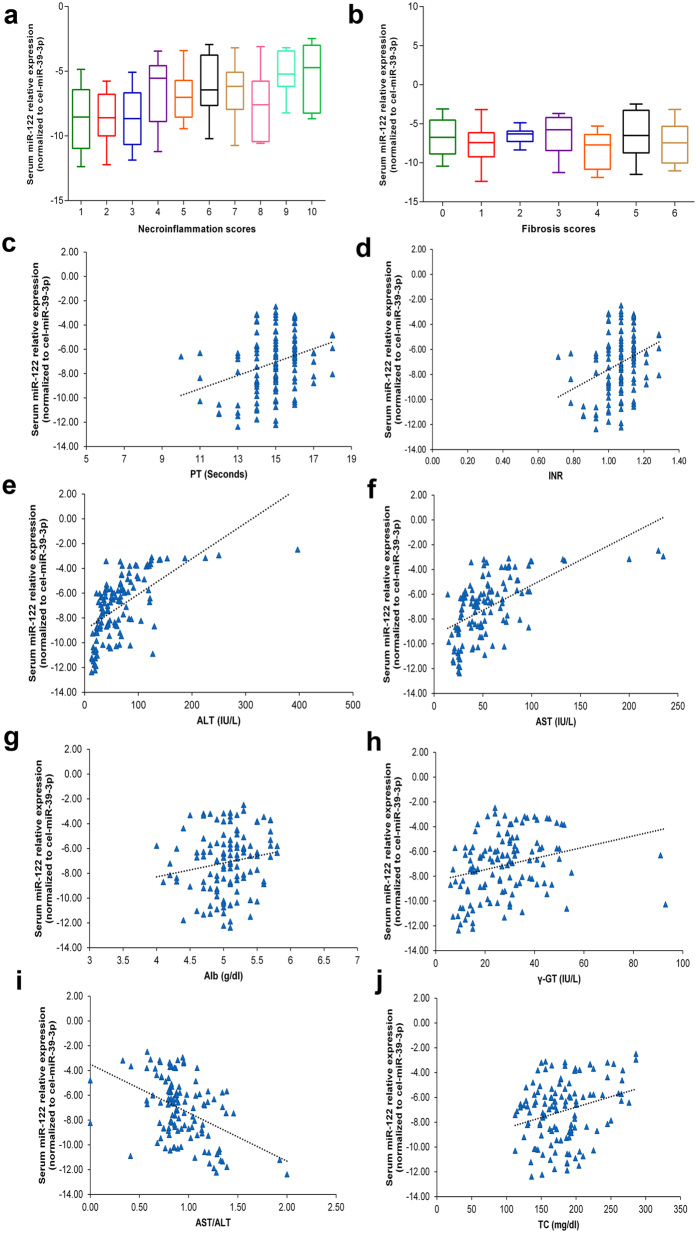
Correlation analysis of serum miR-122. The expression levels of serum miR-122 were significantly correlated with the following clinicopathological features in chronic hepatitis C patients: (**a**) Necroinflammatory scores. (**b**) Fibrosis scores. (**c**) Prothrombin time (PT). (**d**) International normalized ratio (INR). (**e**) Alanine transaminase (ALT). (**f**) Aspartate transaminase (AST). (**g**) Albumin (Alb). (**h**) Gamma-glutamyl transferase (γ-GT). (**i**) AST/ALT ratio, and (**j**) Total cholesterol (TC).

To evaluate whether serum miR-122 levels have potential as a non-invasive diagnostic biomarker, correlation analysis was performed between serum miR-122 levels and the biochemical and histopathological parameters of CHC patients. Serum miR-122 levels were found to be positively correlated with necroinflammation grading scores (*r* = 0.407, *P* = 3.0 × 10^−6^, 95% CI: 0.248 to 0.545) (Fig. 3a). In contrast to hepatic miR-122 levels, serum miR-122 levels showed no significant correlation with fibrosis scores (*r* = −0.03, *P* > 0.05). In the case of liver function parameters, serum miR-122 levels were significantly positively correlated with PT (*r* = 0.325, *P* = 2.0 × 10^−4^, 95% CI: 0.157 to 0.475), INR (*r* = 0.323, *P* = 3.0 × 10^−4^, 95% CI: 0.157 to 0.477), ALT (*r* = 0.684, *P* = 2.70 × 10^−18^, 95% CI: 0.577 to 0.768), AST (*r* = 0.661, *P* = 1.56 × 10^−16^, 95% CI: 0.547 to 0.751), albumin (Alb) (*r* = 0.185, *P* = 0.043, 95% CI: 0.006 to 0.352), γ-GT (*r* = 0.351, *P* = 7.8 × 10^−5^, 95% CI: 0.184 to 0.499) and significantly negatively correlated with the AST/ALT ratio (*r* = −0.408, *P* = 3.0 × 10^−6^, 95% CI: −0.545 to −0.248). In the case of lipid function parameters, a weakly positive significant correlation was noted between serum miR-122 and total cholesterol (TC) levels (*r* = 0.215, *P* = 0.017, 95% CI: 0.039 to 0.378) (Fig. 3c–j).

### Pre-treatment hepatic and serum miR-122 levels correlate with ETR in CHC patients

**Figure 4:**
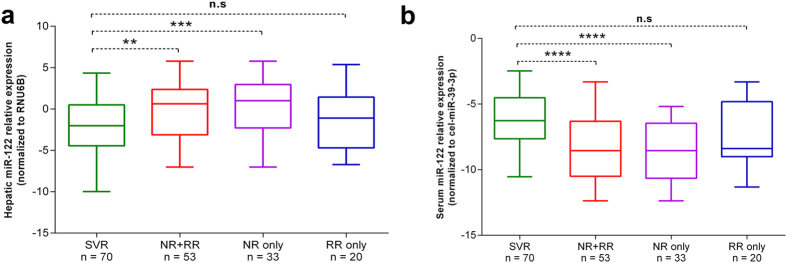
Expression levels of hepatic and serum miR-122 in sustained virological responder (SVR) and non-responder (NR) groups. The differences in expression levels of hepatic (**a**) and serum (**b**) miR-122 were computed between SVR and NR groups. Boxes represent range, median and quartiles of the normalized miR-122 expression (∆Cq) levels. Asterisks indicate level of significant difference between analysed groups and are as follows: ****P < 0.0001, ***P < 0.001, **P < 0.01 and *P < 0.05.

To determine whether pre-treatment levels of hepatic and serum miR-122 correlate with, and potentially serve as a predictive marker of, ETR to PEG-INF/RBV therapy in CHC patients, CHC patients were divided into responder (SVR) (n = 70) and non-responder (NR+RR) (n = 53) groups according to their ETR status (Table 1). We found that pre-treatment levels of hepatic miR-122 were significantly down-regulated in the SVR group in comparison with NR+RR (*P* = 0.01) and NR only (*P* = 0.002) groups (Fig. 4a). In contrast to this, pre-treatment serum miR-122 levels were significantly higher in the SVR group compared with that in the NR+RR (*P* = 0.000030) and NR only (*P* = 0.000008) groups (Fig. 4b). However, there was no significant difference of either hepatic and serum miR-122 levels between SVR and RR only groups (*P* > 0.05). Furthermore, age and gender adjusted univariate and multivariate regression analyses were performed to determine the potential of miR-122 levels, and the clinical features of CHC patients, as predictors of ETR. In univariate analysis, both hepatic and serum miR-122 levels, platelet count, PT, ALT, AST, alkaline phosphatase (ALP), TP, Alb, high-density lipoprotein (HDL) and low density lipoprotein (LDL) were identified as predictors of ETR. However, under multivariate analysis, only serum miR-122 and ALP remained independently associated with ETR (Table 2).

### The diagnostic and prognostic potential of serum miRNA-122 in CHC patients

**Figure 5:**
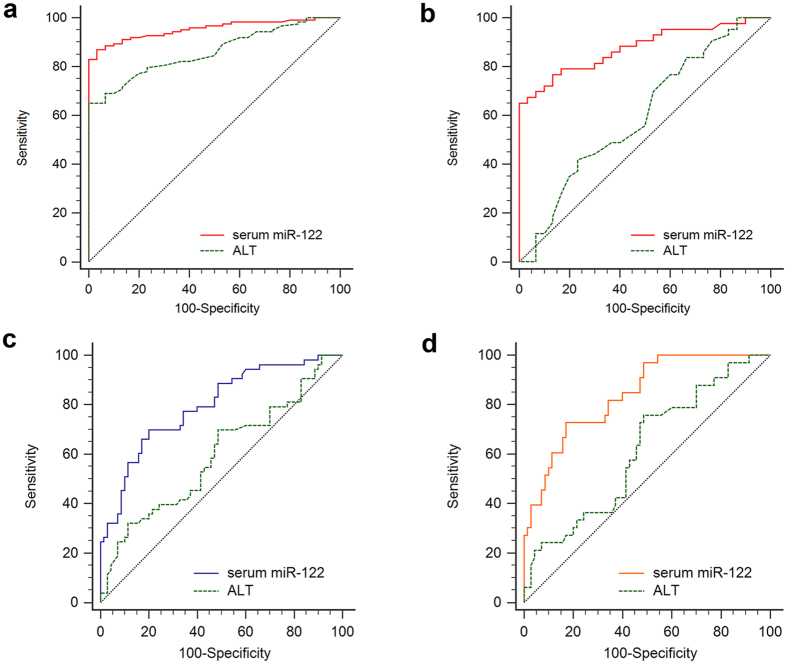
Receiver operator characteristic (ROC) curve analysis. ROC curves were drawn and the area under the curve (AUC) was calculated to evaluate and compare the diagnostic and prognostic potential of serum miR-122 and alanine transaminase (ALT) levels in (**a**) chronic hepatitis C (CHC) patients (normal plus elevated ALT group) versus healthy controls, (**b**) CHC patients (normal ALT group) versus healthy controls, (**c**) SVR group versus NR+RR group, and in the (**d**) SVR group versus NR only group.

To evaluate the diagnostic potential and discriminatory accuracy of serum miR-122 in CHC patients, receiver operator characteristic (ROC) curve analysis was performed. It was observed that serum miR-122 had a robust potential to discriminate CHC patients (including both normal and elevated ALT patients) from healthy controls with an area under the curve (AUC) of 0.954 (95% CI: 0.907–0.981; *P* < 0.0001). At the cut-off value of −10.32, miR-122 had sensitivity and specificity ratio of 87.0% and 96.7% respectively. This is superior to that of ALT which at cut-off value of 40, had an AUC of 0.860 (95% CI: 0.795–0.911; *P* < 0.001) and sensitivity and specificity ratios of 65.0% and 93.3%, respectively (Fig. 5a). Interestingly, serum miR-122 maintained its differentiating potential between CHC patients with normal ALT levels and healthy controls with an AUC of 0.877 (95% CI: 0.779–0.942; *P* < 0.001; cut-off value: −10.32; sensitivity: specificity ratio: 67.4: 96.7%) (Fig. 5b). In contrast to this, ALT levels failed to demonstrate any significant differentiating potential between CHC patients with normal ALT levels and healthy controls (AUC: 0.601; 95% CI: 0.479–0.714; *P* > 0.05) (Fig. 5b). Furthermore, ROC curve analysis for the prognostic significance of serum miR-122 in the SVR and NR+RR groups showed that miR-122 had an AUC of 0.796 (95% CI: 0.714–0.863; *P* < 0.0001; cut-off value: −7.79; sensitivity: specificity ratios: 69.8: 80.0%) (Fig. 5c). In case of SVR and NR only groups, an increase in predictive potential of serum miR-122 was noted with an AUC of 0.840 (95% CI: 0.755–0.905; *P* < 0.0001; cut-off value: −8.18; sensitivity: specificity ratios: 72.7: 89.9%) (Fig. 5d). In contrast to this, ALT showed no significant prognostic value in case of SVR versus NR+RR (*P* > 0.05) (Fig. 5c) and SVR versus NR only (*P* > 0.05) (Fig. 5d) group of patients, indicating overall superiority of serum miR-122 levels in differentiating responders from non-responders.

## Discussion

Since the discovery of the miR-122–HCV interaction mechanism, suggesting that miR-122 facilitates the replication of HCV by binding to its two conserved sites^18^,^32^, investigation into the role of miRNAs in HCV-associated infections has gained much attention as a potential therapeutic strategy. The development of efficient diagnostic markers has always been a focus of the scientific community, and it is not surprising that the suitability of miR-122 as potential biomarker for HCV and its progression have been investigated in various labs worldwide. Although, the majority of these studies have shown that miR-122 levels are deregulated in HCV-associated infections, there are still conflicting results, which warrant further investigations. We propose that these discrepancies could be attributed to the influence of the different viral and host factors and overall experimental designs, particularly miRNA quantification methods, of those studies. Therefore, in the present study we aimed to address these issues as much as possible, with a focus on the Pakistani population, which is currently estimated to have an HCV disease prevalence of 10%. The most prevalent HCV genotype in the Pakistani population is 3, therefore the patients included in this study were of this genotype. First, we quantified the levels of miR-122 in matched tissue and serum samples of the same patients. We then investigated the potential correlations between hepatic and serum miR-122 levels, clinicopathological features, and ETR to PEG-INF/RBV therapy.

It has previously been reported that miR-122 levels are down-regulated in liver tissues infected with HCV genotype 1 and 2^33^. We also found down-regulation of hepatic miR-122 levels in liver tissues of CHC patients infected with HCV genotype 3 as compared with those of healthy controls. This ratio of down-regulation of hepatic miR-122 was more profound and significant in CHC patients with higher ALT levels, increased necroinflammation, and fibrosis compared with those with comparatively lower ALT levels and with little, or moderate, liver damage. This indicates that a decrease in hepatic miR-122 levels is potentially correlated with an increase of disease severity in CHC patients. Although the exact mechanism of the decrease in miR-122 is not yet known in the case of liver damage, it has previously been proposed that miR-122, which is synthesized in hepatocytes, releases into the blood stream as a consequence of liver injury and/or a persistent inflammatory state^34^. The findings of a correlation analysis between hepatic miR-122 and clinicopathological features also favours the above described miR-122 release model, as hepatic miR-122 levels were found to be significantly associated with liver function parameters and histopathological scores in our study cohort. In contrast to our and previously reported decreased hepatic miR-122 levels, no significant differences were observed in hepatic miR-122 levels between CHC patients and controls by Bostjancic *et al.*^35^. We suspect that this difference could be due to two possible reasons; (i) only 15 HCV genotype 3 liver tissues were present out of a total of 74 liver tissues, (ii) the total RNA extraction was performed from formalin-fixed, paraffin embedded (FFPE) liver tissues of CHC patients which had been already used for histological analyses. The control samples were obtained from histologically normal surgical margins of hepatocellular carcinoma (HCC) patients and stored in RNAlater. Although miRNAs have been reported to be comparatively stable in FFPE samples, a long term assessment of potential differences in expression patterns between FFPE and liver biopsies stored in other preservative agents such as RNAlater is not yet available. Moreover, in our opinion, the control tissues cannot be referred to as “true controls”. Even though they were taken from non HCV-HCC patients, there is no information given on whether they were also negative for any other etiology, as hepatic miR-122 levels also tend to deregulate in case of HBV and non-viral HCC. Hence, at this stage, it is not clear whether observed differences in hepatic miR-122 levels were due to some actual biological aspect or the influence of the choice of samples selection and preservation approaches. Similarly, in another recent report by Oliveria *et al.*^36^ in which the authors used FFPE samples from HCV genotype 3 liver tissues, hepatic miR-122 levels were found to be up-regulated. However, their findings of up-regulated miR-122 cannot be compared directly with our data as there were no healthy controls included in their study design and the analysis was focused only on comparing miR-122 expression levels between patients of two different HCV genotypes.

Recently, miRNAs have gained much attention as potential non-invasive diagnostic biomarkers due to their stable nature and constant expression in easily accessible serum samples, rather than biopsied organ samples. Therefore, whether serum miRNAs serve as suitable markers for HCV-infections is an important question. An initial study found that mouse serum miRNA-122 levels elevate in response to drug induced liver injury and this was proposed as a potential biomarker of liver damage^37^. Shortly after that Bihrer *et al.*^31^ expression profiled serum miRNA-122 in CHC patients (genotypes 1 and 2) and investigated their correlation with various clinical parameters. The authors also observed a significant up-regulation of serum miR-122 and a significant correlation with elevated ALT and inflammatory scores. Whether the same holds true for genotype 3 CHC patients has not been evaluated before on a large scale. Therefore, in order to further explore the feasibility of miR-122 as a diagnostic biomarker in our study cohort, we investigated correlations between serum and hepatic miR-122 levels and clinicopathological features. Based on the miR-122 release model, serum miR-122 levels are expected to rise with an increase in severity of disease and a decrease in hepatic miR-122 levels. A significant inverse correlation was noted in our study of miR-122 levels between matched tissue and serum samples of CHC patients, which favoured this hypothesis. Another interesting finding was that serum miR-122 levels were significantly higher in genotype 3 CHC patients with either normal or elevated ALT levels compared with those in controls, indicating that miR-122 levels could be potentially more sensitive to HCV-induced liver damage than ALT. In agreement with our data, the ability of serum miR-122 to discriminate between normal and elevated ALT levels in genotype 1 CHC patients has also been reported previously^28^. While this manuscript was in preparation, Zhang *et al.*^38^ reported serum miRNA profiling in 49 CHC patients, the majority of which were infected with genotype 1 (n = 37). The findings of Zhang *et al.* corroborate our data as they also noted significant up-regulation of serum miR-122 in CHC patients versus controls and significant positive correlations with ALT and AST levels. Overall, this indicates that serum miR-122 has the potential to serve as a biomarker in case of CHC patients. However, some exceptions to above studies have also been noted. For instance, Wang *et al.*^29^ did not observe any correlation of serum miR-122 with any of the clinical parameters including ALT and histopathology findings of HCV genotype 1 CHC patients^29^. Similarly, in another study, miR-122 levels were not significant when compared between control and genotype 1 CHC patients with normal ALT levels^31^. However, in the latter study, ALT levels were not given for the healthy group. As mentioned above, we suspect that such differences in findings can be somewhat attributed to differences in sample size, host and viral factors and expression profiling methods and warrants further investigations with a larger cohort.

Interestingly, in our data it was also observed that the majority of the clinicopathological features that were directly correlated with hepatic miR-122 levels were inversely correlated with serum miR-122 levels, with few exceptions. For instance, hepatic miR-122 levels were significantly correlated with fibrosis scores, whereas no correlation was observed between fibrosis and serum miR-122 levels. This indicates a potentially low significance of serum miR-122 as a fibrosis specific biomarker in CHC patients. The lack of correlation between serum miR-122 and fibrosis has also been reported previously in genotype 1 CHC patients^31^ which is in agreement to our data. Significant correlations between serum miR-122 and major liver function parameters, including ALT, AST, γ-GT, Alb, and necroinflammation scores, as observed in the present study and in majority of previous studies^28^,^31^,^38^, collectively suggest that serum miR-122 could be a more suitable marker for progressive liver damage and inflammation.

In addition to the association of miR-122 with HCV, it is also known that miR-122 regulates several genes from different metabolic pathways, including cholesterol biosynthesis. In previous knockdown studies, it has been shown that the inhibition of miR-122 expression leads to reduced cholesterol biosynthesis and an overall 25–30% reduction in TC levels without any signs of toxicity, suggesting it as a potential therapeutic target for hypercholesterolemia^19^,^39^. Deregulated cholesterol biosynthesis is also very evident in the case of CHC infections. Therefore, we performed correlation analyses on hepatic and serum miR-122 levels and lipid related features. No significant correlation was observed between hepatic miR-122 and lipid profile parameters. However, serum miR-122 levels showed a significant direct correlation with TC levels. Although correlations between lipid profile parameters and serum miR-122 levels have been recently reported in non-alcoholic fatty liver disease (NAFLD) and hyperlipidaemia^40^, to our knowledge this is the first study to report a significant correlation between TC and serum miR-122 levels in CHC patients. It is interesting to note that in a previous study no correlation between any lipid profile parameters and serum miR-122 levels in genotype 1 CHC patients was found^26^. Whether this could be a genotype-specific observation warrants further investigation.

The prediction of ETR to PEG-INF/RBV has always been an important aspect of HCV research. In this regard, several host and viral factors have been analysed in different populations and experimental design conditions to determine such potential predictors. The most notable example in this regard is IL-28B, whose polymorphisms have been reported to associate with ETR. However, the available IL-28B data is still somewhat conflicting^41^,^42^ and to date, no universally applicable predictive marker of PEG-INF/RBV therapy has been established. Due to the significance of miR-122 in HCV infections, the association between hepatic miR-122 expression and ETR has been evaluated in some recent reports. For instance, pre-treatment expression of hepatic miR-122 has been reported to be decreased in NR compared with SVR patients of HCV genotypes 1 and 2^43^. Conversely, Estrabaud *et al.*^25^ observed no correlation between pre-treatment decreased levels of hepatic miR-122 and ETR. In contrast, we observed a significant up-regulation of hepatic miR-122 levels in NR patients compared with those who achieved SVR status. The reason for this difference could be due to the fact that both of above mentioned studies had a majority of samples from HCV genotypes other than 3. Although in the later study there were 14 HCV genotype 3 samples out of a total 133 HCV patient samples, we feel this number is not large enough to influence the overall expression analysis, as their study samples were dominated by HCV genotype 1 (n = 80). The significant correlation of ETR with hepatic miR-122 levels indicates that it could serve as a predictive marker of ETR in CHC patients. However, measuring hepatic miR-122 levels demands an invasive procedure of liver biopsy and is not widely feasible. As a significant correlation was observed between hepatic and serum miR-122 levels in our study, we compared serum miR-122 levels in CHC patients according to their ETR. Interestingly, pre-treatment serum miR-122 levels were found to be significantly up-regulated in SVR compared with those in NR+RR and NR only group of patients, which was also further validated via logistic regression analysis showing that serum miR-122 levels remained independently associated with ETR in the SVR group of patients. Up-regulation of serum miR-122 levels in the SVR group in comparison with those in the NR group has also been reported previously in HCV genotype 1-, 2- and 4-infected Taiwanese and Egyptian patients, respectively^26^,^44^. However, unlike our study, neither of these studies analysed hepatic miR-122 levels in relation to ETR in matched liver tissues, so it is not known what correlation hepatic miR-122 might have with ETR in these studies. Overall, we propose that elevated pre-treatment serum miR-122 levels have the potential to predict ETR in genotype 3 CHC patients.

The differences in deregulated levels of both hepatic and serum miR-122 levels between CHC patients and controls may be attributed to HCV infection. However, before suggesting miR-122 as a potential biomarker, we were interested to determine the level of sensitivity and specificity miR-122 offers compared with other biochemical factors, such as ALT, in CHC patients that also show deregulation after HCV infection. To achieve this, ROC curve analysis was performed. As the focus was to elucidate a non-invasive diagnostic biomarker potential of miR-122, ROC curves were constructed for serum miR-122, which was found to be superior to ALT in discriminating CHC patients from healthy controls. Interestingly, serum miR-122 also maintained its ability to differentiate between CHC patients with normal ALT levels and healthy controls. In agreement with logistic regression analysis, serum miR-122 ROC curve analysis between SVR: NR+RR and SVR: NR only groups also revealed its prognostic potential to discriminate between these groups. On the other hand, ALT levels were not significantly associated with SVR. This is in agreement to previous studies which also demonstrated lack of prognostic value of ALT levels with ETR in CHC patients of genotype 3 and 1^45^,^46^.

In summary, the results of the present study suggest that circulating levels of liver specific miR-122 reflect changes in hepatic miR-122 concentrations due to HCV infections. Serum miR-122 levels seem to be a sensitive predictor of disease progression and may possess more potential than the routinely performed biochemical tests for monitoring HCV-associated liver damage. In addition to this, miR-122 was also found to be an interesting and worthy of further evaluation predictor of ETR. Although, it was beyond the scope of this study, it would be interesting to perform IL-28B genotyping on CHC genotype 3 patients, in parallel with miR-122 expression profiling. This would allow comparison of the predictive power of both, as IL-28B polymorphisms have also been reported as a potential predictor of ETR in CHC patients. Studies are currently underway in our labs to investigate this idea. Overall, we propose that miR-122 holds promising potential to be considered for use in clinical practice following similar validation studies in larger cohorts of patients, preferably with matched tissue and serum samples.

## Materials and Methods

Due to space constraints, please refer to the Supplementary Data S1 online for detailed description of study design, study subjects, samples collection, virological assays and histology analysis.

### Ethics statement

The study protocol was approved by the Ethics Committee of the Centre of Excellence in Molecular Biology (CEMB), University of the Punjab, Lahore, Pakistan. Written informed consent for the use of biological samples and clinical records was given by all patients who participated in this study. The study was conducted in accordance with the ethical guidelines of the 1975 Declaration of Helsinki and the International Conference on Harmonization Guidelines for Good Clinical Practice.

### Total RNA isolation

#### From liver tissues

Total RNA including miRNA was extracted from control and CHC-positive liver tissues using a miRNeasy mini extraction kit (Qiagen, Valencia, CA, USA) following some modifications according to the tissue weight. First, the liver tissues stored in RNAlater at −80 °C were allowed to thaw at room temperature (15–20 °C) for 5 min. A fixed amount of liver tissue (3 mg) was excised from each sample using disposable sterile surgical blades and submerged into 2-ml LoBind tubes, which were pre-filled with 400 μl of QIAZOL lysis from the miRNeasy mini kit. Each sample was separately homogenized using a hand-held Tissue Master 125 tissue homogenizer (Omni International, Marietta, Georgia, USA) fixed with a sterile 5-mm stainless steel probe until no visible particles are seen. The remaining 400 μl lysis solution was then added for a final volume of 800 μl of lysis reagent, which was left at room temperature for 5 min to promote complete dissociation of nucleoprotein complexes. After this, 200 μl of chloroform was added followed by vortexing for 20 s, and incubation at room temperature for 3 min. The LoBind tubes containing homogenate were then centrifuged at 14,000 rpm at 4 °C for 15 min for phase separation. The upper transparent aqueous phase was transferred to new 2-ml LoBind tubes and 100% ethanol was added to maintain the manufacturer’s recommended 1.5 volume ratio between the collected upper aqueous phase and ethanol, and mixed thoroughly by pipetting. The miRNeasy mini spin columns were placed into 2-ml collection tubes (supplied with the kit) and 700 μl of aqueous phase plus ethanol mixture was pipetted into the spin column and centrifuged at 10,000 rpm at room temperature for 15 s. The same procedure was repeated until all of the aqueous phase plus ethanol mixture was passed through the mini columns. During washing, 700 μl of buffer RWT was added to mini spin columns fixed into new collection tubes and centrifuged at 10,000 rpm at room temperature for 15 s. Two washing with 500 μl of RPE buffer was then performed at 10,000 rpm at room temperature for 15 s and 2 min respectively to avoid ethanol carryover. To elute the total RNA including miRNAs, the spin columns were placed into 1.5-ml LoBind collection tubes. A fixed volume (35 μl) of DNAse/RNAse-free water was pipetted directly onto the middle of spin column membrane and the column was centrifuged at 10,000 rpm for 1 min at room temperature. The quality and integrity of the total RNA was assessed using NanoDrop 8000 spectrophotometer (NanoDrop Technologies, Wilmington, DE, USA) and Agilent 2100 Bioanalyzer (Agilent Technologies, Santa Clara, CA, USA).

#### From serum

Total RNA containing miRNA was extracted from the serum samples using a miRNeasy mini kit and following the manufacturer’s supplementary protocol for the isolation of miRNA from serum samples. Briefly, 200 μl of serum sample was mixed with 1000 μl QIAzol lysis reagent (1:5) in 2-ml LoBind tubes and vortexed vigorously for 5 min. The tubes containing homogenate were then placed at room temperature for 5 min for the complete dissociation of nucleoprotein complexes. In order to monitor extraction efficacy and for the minimization of sample-to-sample variation, 5 ul of 5 nM of synthetic *Caenorhabditis elegans* miRNA-39 (cel-miR-39-3p) miScript mimic was added to each lysed sample. This step was followed by the addition of 200 μl of chloroform and vigorous vortexing for 1 min and placed at room temperature for 5 min. The samples were centrifuged at 12,000 rpm at 4 °C for 15 min for phase separation and the upper aqueous phase transferred to new 2-ml LoBind tubes. According to the volume of aqueous phase obtained, 1.5 volumes of 100% ethanol was added and mixed thoroughly by pipetting up and down several times. The washing steps were performed as given above for tissue samples. After the washing steps, the spin columns were placed into collection tubes and the total RNA including miRNAs was eluted in 35 μl DNAse/RNase-free water via centrifugation for 1 min at 10,000 rpm and immediately stored at −80 °C until further use.

### Reverse transcription

Total RNA from tissue and serum samples was reverse transcribed using miScript II RT Kit (Qiagen, Valencia, CA, USA) following the manufacturer’s instructions. A fixed amount of 1 μg and fixed volume of 5 μl of total RNA from tissue and serum samples respectively was reverse transcribed in a final mixture of 20 μl containing 4 μl 5× miScript HiSpec Buffer, 2 μl 10× Nucleics mix, 2 μl miScript Reverse Transcriptase Mix and RNase-free water. The volume of RNase-free water was variable and adjusted according to the concentration of 1 μg of the eluted total RNA for each tissue sample. In the case of serum samples, 7 μl of RNase-free water was used. The reaction was then incubated at 37 °C for 60 min followed by 95 °C for 5 min on a thermocycler (Applied Biosystems, Foster City, CA). The synthesized undiluted cDNAs were then immediately stored in LoBind tubes at −20 °C until further use.

#### Real-time quantitative PCR

RT-qPCR analysis for tissue and serum samples was performed using miScript SYBR Green PCR Kit (Qiagen, Valencia, CA, USA) following the manufacturer’s instructions. Custom miScript miRNA PCR arrays (Qiagen, Valencia, CA, USA) were used which were provided pre-coated with miScript Primer Assays (Qiagen, Valencia, CA, USA) for hsa-miR-122-5p (assay ID: MS00003416), cel-miR-39-3p (assay ID: MS00019789), and RNU6B (assay ID: MS00033740). RNU6B and cel-miR-39 were used as normalization controls for miR-122 levels in tissue and serum samples respectively. As there is currently no consensus on a suitable normalization control for serum miRNA profiling, and cel-miR-39 has been shown to serve as a stable reference normalization control, we also used it as normalization control in the present study. First, the 20 μl cDNA template was diluted by adding 200 μl RNase-free water to ensure a final concentration of 1 ng per tube/well. A reaction mix of 25 μl per sample was then prepared as follows: 12.5 μl 2× QuantiTect SYBR Green PCR Master Mix, 2.5 μl 10× miScript Universal Primer, 2.5 μl diluted cDNA and 7.5 μl RNase-free water. All real-time PCR reactions were performed in duplicate on an iQ5 Cycler Multicolor real-time PCR detection system (Bio-Rad Laboratories, Hercules, CA, USA). The amplification cycling conditions were: 95 °C for 15 min, followed by 40 cycles of 94 °C for 15 s, 55 °C for 30 s and 70 °C for 30 s. The relative expression levels, of hepatic and serum miR-122 were calculated using a 2^∆∆Cq^ method where, ∆∆Cq = ∆Cq (Cq^RNU6B^ − Cq^miR-122^)_CHC_ − ∆Cq (Cq^RNU6B^ − Cq^miR-122^)_Controls_ and ∆∆Cq = ∆Cq (Cq^cel-miR-39^ − Cq^miR-122^)_CHC_ − ∆Cq (Cq^cel-miR-39^ − Cq^miR-122^)_Controls_, respectively. The Cq values refer to the number of quantification cycles required for the fluorescent signal to cross the defined threshold level in RT-qPCR and are inversely proportional to the expression level of the miRNA in question. Therefore, the lower the Cq value, the higher the expression level of miRNAs and vice versa. For the ∆Cq values to reflect actual/direct expression instead of the opposite, a modification of the Livak method was used where the Cq values of normalizers were subtracted from that of miR-122, instead of subtracting the Cq of miR-122 from the Cq of normalization controls. All RT-qPCR reactions were performed in accordance to the Minimum Information for Publication of Quantitative Real-Time PCR Experiments (MIQE) guidelines^47^.

### Statistical analyses

The differences between two or more groups were evaluated using the Mann–Whitney *U* or the Kruskal–Wallis tests, respectively. A Spearman non-parametric rank test was used to determine the correlations, computed as the correlation coefficient *r*, between the expression levels of hepatic and serum miR-122 and the clinicopathological parameters. Univariate and multivariate logistic regression analyses were performed to determine the association between hepatic and serum miR-122 levels, clinicopathological features, and with ETR. Statistical analyses were performed using SPSS software version 23 (SPSS Inc., Chicago, IL, USA). ROC curves were generated to determine the diagnostic potential of serum miR-122 via calculation of the AUC, sensitivity, and specificity according to standard formulas using MedCalc Statistical Software version 15.8 (MedCalc Software, Ostend, Belgium). *P* values were two-sided, and values less than 0.05 were considered to be statistically significant.

## Additional Information

**How to cite this article**: Butt, A. M. *et al.* Parallel expression profiling of hepatic and serum microRNA-122 associated with clinical features and treatment responses in chronic hepatitis C patients. *Sci. Rep.* **6**, 21510; doi: 10.1038/srep21510 (2016).

## Supplementary Material

Supplementary Information

## Figures and Tables

**Table 1:** Demographics and clinical features of study participants.

Parameters^a^	Chronic Hepatitis C Patients (Genotype 3)				Controls (n = 60)
*General features*	Total (n = 123)	SVR (n = 70)	NR^b^ (n = 53)	RR^c^ (n = 20)	
Age (years)	32.7 ± 9.9 (18–60)	32.3 ± 8.4 (18–50)	33 ± 11.3 (18–60)	33.9 ± 13.9 (18–60)	39.2 ± 12.9 (20–61)
Gender (M/F)	61/62	35/35	26/27	12/08	30/30
Platelet count (× 10^3^/μl)	220 ± 69.4 (110–426)	209.4 ± 65.9 (110–402)	231.7 ± 71.1 (151–426)	227.8 ± 65.9 (151–426)	246.9 ± 69.5 (160–400)
*Liver function indicators*					
BT (mg/dl)	0.7 ± 0.3 (0.4–1.6)	0.8 ± 0.3 (0.4–1.5)	0.7 ± 0.2 (0.4–1.6)	0.7 ± 0.2 (0.5–1.1)	0.6 ± 0.1 (0.4–0.9)
BD (mg/dl)	0.4 ± 0.2 (0.1–0.8)	0.4 ± 0.2 (0.1–0.8)	0.3 ± 0.1 (0.2–0.6)	0.3 ± 0.1 (0.2–0.6)	0.2 ± 0.1 (0.1–0.3)
BI (mg/dl)	0.4 ± 0.2 (0.2–1)	0.4 ± 0.1 (0.2–0.8)	0.4 ± 0.2 (0.2–1)	0.4 ± 0.1 (0.2–0.6)	0.4 ± 0.1 (0.2–0.7)
ALT (IU/L)	65 ± 51.7 (13–397)	73.6 ± 58.9 (13–397)	55.1 ± 39.6 (13–187)	71.2 ± 51 (14.5–187)	22.6 ± 8.2 (8–40)
AST (IU/L)	54.8 ± 37.5 (14–235)	62.4 ± 43.3 (14–235)	45.7 ± 26.3 (19.3–134)	53.6 ± 35.1 (19.3–134)	27.2 ± 7.4 (14–44)
AST/ALT ratio	0.9 ± 0.3 (0.0–2.0)	0.9 ± 0.3 (0.6–2.0)	0.9 ± 0.3 (0.0–2.0)	0.9 ± 0.3 (0.0–1.4)	0.6 ± 0.1 (0.0–0.7)
ALP (IU/L)	236.5 ± 46.4 (137.7–325)	220.4 ± 39.9 (137.7–325)	245 ± 46 (166–311)	237.5 ± 47.3 (171–305)	194 ± 37.4 (90–240)
TP (g/dl)	7.7 ± 0.4 (6.8–8.9)	7.8 ± 0.4 (6.8–8.9)	7.5 ± 0.4 (6.9–8.7)	7.5 ± 0.4 (6.9–8.2)	6.9 ± 0.3 (6.4–7.6)
Alb (g/dl)	5.1 ± 0.4 (4.0–5.8)	5.1 ± 0.3 (4.3–5.7)	5 ± 0.4 (4–5.8)	4.9 ± 0.4 (4–5.8)	4.1 ± 0.2 (3.8–4.5)
Glb (g/dl)	2.6 ± 0.4 (1.5–3.7)	2.7 ± 0.4 (1.5–3.7)	2.6 ± 0.4 (1.8–3.7)	2.6 ± 0.5 (1.2–3)	2.8 ± 0.3 (2.5–3.5)
A/G ratio	2 ± 0.4 (1.2–3.5)	2 ± 0.5 (1.3–3.5)	2 ± 0.4 (1.2–3)	2 ± 0.5 (1.2–3)	1.5 ± 0.2 (1.1–1.7)
γ−GT (IU/L)	28 ± 14.5 (7–91)	25.9 ± 11.7 (7–55)	30.6 ± 16.9 (7–91)	31.3 ± 11.7 (7–53)	23.8 ± 12.3 (6–44)
PT (seconds)	14.8 ± 1.4 (10–18)	15.2 ± 1.2 (11–18)	14.4 ± 1.5 (10–16)	14.7 ± 1 (13–16)	14.5 ± 1.4 (11–16)
APTT (seconds)	36.9 ± 2.1 (32–42)	37.1 ± 2.2 (34–42)	36.5 ± 2 (32–41)	35.7 ± 2.2 (32–40)	37.3 ± 2.7 (33–43)
INR	1.0 ± 0.2 (0.0–1.3)	1.1–0.2 (0.0–1.3)	1 ± 0.2 (0.0–1.1)	1–0.2 (0.0–1.1)	1–0.1 (0.8–1.1)
*Serum lipid measures*					
TC (mg/dl)	181.1 ± 39.5 (112.4–286.0)	179.4 ± 43.6 (112.4–286)	183 ± 34 (115–254)	196.2 ± 33.9 (138–254)	144.7 ± 21.8 (113–197)
TG (mg/dl)	141.3 ± 32.7 (78–250)	137.4 ± 28.2 (78–234)	146 ± 36.9 (97–250)	141.6 ± 31 (110–195)	132.4 ± 48.6 (80–242)
HDL-c (mg/dl)	38 ± 6.2 (26.6–56)	36.2 ± 5.3 (26.6–49.7)	40.2 ± 6.4 (27–56)	43.5 ± 4.5 (33–52.3)	49.4 ± 5.7 (41–60)
LDL-c (mg/dl)	109.5 ± 29.8 (60.4–204)	101.9 ± 24.8 (61.1–189.3)	118.5 ± 32.7 (60.4–204)	121.1 ± 34.1 (60.4–175.7)	87.4 ± 22.2 (60–130)
VLDL-c (mg/dl)	28.1 ± 6.2 (15.6–50)	27.6 ± 5.3 (15.6–47)	28.6 ± 7.1 (20–50)	28.4 ± 5.8 (22–39)	17.9 ± 6.5 (4–29)
*Necroinflammation (Grading)*					
Minimal (0–3)	46 (37.4)	22	21	10	–
Mild (4–8)	66 (53.6)	34	32	13	–
Moderate (9–13)	11 (8.9)	10	4	3	–
Severe (14–18)	0	0	0	0	–
*Fibrosis (Staging)*					
Low stage fibrosis (0–3)	95 (77.2)	46	41	16	–
High stage fibrosis (4–6)	28 (22.7)	20	16	10	–
*Virus characteristics*					
Viral load (log IU/ml)	5.3 ± 0.9 (3.6–7.9)	5.3 ± 0.9 (3.6–7.9)	5.2 ± 0.9 (4.1–6.8)	5 ± 1 (4.1–6.8)	–
*Sugar levels profile*					
Fasting glucose (mg/dl)	68 ± 10 (55–85)	69.5 ± 10.3 (55–89)	69.8 ± 15.8 (55–123)	77.1 ± 19.2 (58–123)	89.4 ± 12.5 (70–108)
Fasting Insulin (mIU/L)	13.54 ± 3.2 (6.9–20.3)	14.2 ± 3.5 (6.9–20.3)	14.5 ± 2 (12.1–18)	14.5 ± 1.7 (12.1–16)	8.6 ± 1.3 (7–11)
HOMA-IR	2.4 ± 0.5 (1.3–3.3)	2.4 ± 0.5 (1.3–3.7)	2.5 ± 0.6 (1.7–4.2)	2.7 ± 0.6 (2.2–4.2)	1.9 ± 0.1 (1.7–2.0)

Clinical parameters are given as mean ± SD (range) of no.(%) of patients.

^a^clinical parameters.

^b^complete non-responders including relapsers.

^c^relapsers only excluding non-responders; M, male; F, female; BT, bilirubin total; BD, bilirubin direct; BI, bilirubin indirect; ALT, alanine aminotransferase; AST, aspartate aminotransferase; ALP, alkaline phosphatase; TP, total proteins; Alb, albumin; Glb, globulin; γ-GT, gamma-glutamyl transferase; PT, prothrombin time; APTT, activated partial thromboplastin time; INR, international normalized ratio; TC, total cholesterol; TG, triglycerides; HDL-c, high density lipoprotein cholesterol; LDL-c, low density lipoprotein cholesterol; VLDL-c, very low density lipoprotein cholesterol; SVR, sustained virological responder; NR, non-responder; RR, relapser. Please refer to the Supplementary Data S1 online for reference ranges of biochemical tests.

**Table 2:** Logistic regression analysis of predictors of sustained virological response to PEG-INF/RBV therapy in CHC patients.

*Variables*	Univariate					Multivariate				
	B	S.E	*P*	OR	95% CI	B	S.E	*P*	OR	95% CI
Hepatic miR-122	−0.137	0.055	0.014*	0.872	0.782–0.972					
Serum miR-122	0.355	0.088	0.000057*	1.426	1.200–1.695	0.918	0.433	0.003*	2.505	1.071–5.855
Age (years)	−0.009	0.018	0.639	0.991	0.956–1.028					
Gender (M/F)	−0.038	0.364	0.917	0.963	0.472–1.966					
Platelets count	−3.112	1.518	0.040*	0.045	0.002–0.873					
ALT	2.490	0.721	0.000549*	12.059	2.938–49.505					
AST	3.435	0.960	0.000345*	31.046	4.731–203.720					
ALP	−0.015	0.007	0.029*	0.985	0.971–0.998	−0.029	0.012	0.016*	0.972	0.949–0.995
TP	1.362	0.473	0.004*	3.904	1.544–9.873					
Alb	1.395	0.545	0.010*	4.037	1.387–11.752					
PT	0.305	0.138	0.026*	1.356	1.036–1.776					
HDL-c	−0.105	0.034	0.002*	0.900	0.842–0.963					
LDL-c	−4.182	1.688	0.013*	0.015	0.001–0.418					

* represents *P* < 0.05; S.E, standard error; CI, confidence interval; OR, odds ratio; ALT, alanine aminotransferase; AST, aspartate aminotransferase; ALP, alkaline phosphatase; TP, total proteins; Alb, albumin; Glb, globulin; γ-GT, gamma-glutamyl transferase; PT, prothrombin time; HDL-c, high-density lipoprotein cholesterol; LDL-c, low density lipoprotein cholesterol.
