# Supplementary material for: Parallel expression profiling of hepatic and serum microRNA-122 associated with clinical features and treatment responses in chronic hepatitis C patients
Source: Sci Rep. 2016 Feb 22;6:21510. doi: 10.1038/srep21510 (PMC4761907; doi:10.1038/srep21510)
Supplement: Supplementary Information [file srep21510-s1.doc]

# Supplementary Data S1 Online

# Parallel expression profiling of hepatic and serum microRNA-122 associated with clinical features and treatment responses in chronic hepatitis C patients

Azeem Mehmood Butt^1^, Arsalan Raja^2^, Shafiqa Siddique^3^, Jahangir Sarwar Khan^3^, Muhammad Shahid^1^, Ghias-Un-Nabi Tayyab^2^, Zahid Minhas^4^, Muhammad Umar^4^, Muhammad Idrees^1^,* & Yigang Tong^5^,*

^1^ Molecular Virology Laboratory, Centre of Excellence in Molecular Biology (CEMB), University of the Punjab, Lahore, Pakistan.

^2^ Medicine Unit-I, Department of Medicine, Lahore General Hospital (LGH), Lahore, Pakistan.

^3^ Surgical Unit-I, Department of Surgery, Holy Family Hospital, Rawalpindi, Pakistan.

^4^ Medicine Unit-I, Department of Medicine, Centre for Liver and Digestive Diseases (CLD), Holy Family Hospital, Rawalpindi, Pakistan.

^5^ State Key Laboratory of Pathogen and Biosecurity, Beijing Institute of Microbiology and Epidemiology, Beijing, People's Republic of China.

*Correspondence and requests for materials should be addressed to Y.T. **(**email: tong.yigang@gmail.com) & M.I. (email: idreeskhan@cemb.edu.pk)

# Materials and Methods

## Ethics statement

The study protocol was approved by the Ethics Committee of the Centre of Excellence in Molecular Biology (CEMB), University of the Punjab, Lahore, Pakistan and participating hospitals. Written informed consent for the use of biological samples and clinical records was given by all patients who participated in this study. The study was conducted in accordance with the ethical guidelines of the 1975 Declaration of Helsinki and the International Conference on Harmonization Guidelines for Good Clinical Practice.

**Study design and subjects**

The study cohort comprised a total of 183 participants including 123 CHC patients of HCV genotype 3 and 60 healthy subjects who were enrolled between January 2011 and December 2013. The participants’ characteristics are summarized in Table 1. Inclusion criteria for CHC patients were: (i) treatment-naive and confirmed positive for both anti-HCV antibodies and HCV RNA for at least 6 months via third generation ELISA and HCV detection real-time PCR assays, respectively; (ii) confirmed positive for HCV genotype 3 only via genotyping assays; (iii) biopsy-proven presence of CHC; (iv) no concurrent hepatitis B [seropositive for hepatitis B surface antigen (HBsAg)], human immunodeficiency virus (HIV), or autoimmune hepatitis; (v) no history of drug or alcohol use; and (v) no history of smoking. Controls were included in the study if they had (i) no history of smoking, alcohol intake, or hypertension; (ii) were not on any type of medication; (iii) had within normal range blood tests, liver tests, and lipid profiles; (iv) negative serology for HBV, HCV and HIV; and (v) no history of diabetes mellitus or any kind of liver disease, including non-alcoholic fatty liver disease. Exclusion criteria for both patients and controls were: (i) hepatocellular carcinoma, or splenomegaly in imaging studies, with or without histological confirmation; (ii) history of diabetes mellitus; (iii) co-infection with HBV/HIV; (iv) any type of anti-hypertensive or lipid-lowering medication; and (v) smoking or any kind of drug abuse; and (vi) CHC patients infected with HCV genotypes other than genotype 3 were also excluded.

**Management and classification of patients according to end treatment response**

All CHC patients were treated with PEG-INF/RBV therapy for 24 weeks according to the treatment guidelines for HCV genotype 3 CHC patients[^1^](#_ENREF_1). The end treatment response (ETR) at the end of 24 weeks of PEG-INF/RBV therapy was established on the basis of circulating HCV RNA levels determined via real time PCR. The overall treatment duration for each patient was divided into two phases; (i) therapeutic phase (from week 0 – week 24), and (ii) observational phase (from week 24 – week 48). The patients were then classified as: sustained virological responders (SVR): patients negative for serum HCV RNA levels at the end of the therapeutic phase and re-confirmed as negative for serum HCV RNA levels at the end of observational phase; relapsers (RR): patients whose serum HCV RNA levels remained negative during the therapeutic phase but re-appeared at the end of observational phase; and non-responders (NR): patients whose serum HCV RNA levels remained positive or without any signs of decline throughout the therapeutic phase.

## Sample collection and preparation

***Liver tissues:*** Liver biopsy samples were taken from CHC patients using the ultrasound-guided ACECUT 16G automatic biopsy gun (TSK Laboratory, Tochigi, Japan) by a gastroenterologist in the presence of a radiologist 1 week before commencing PEG-INF/RBV treatment. The tissue sample was divided into two equal portions and immediately submerged into RNAlater (Life Technologies, Carlsbad, CA, USA) and a tissue fixation agent for total RNA isolation and histopathology analyses respectively. Samples in RNAlater were incubated overnight at 4 °C and then stored at −80 °C until further use. The control tissues were obtained from HCV-/HBV-/HIV- individuals during surgical procedures. Control tissues were also stored in RNAlater at −80 °C until further use.

***Serum:*** Ten millilitres of peripheral blood was collected from treatment-naive CHC patients after overnight fasting directly into gel-activated serum separator tubes (Becton-Dickinson, Oxford, UK) 1 day before liver biopsy. A strict “needle to freeze time” was maintained for every sample. The collection tubes were allowed to remain at room temperature for 20 min to ensure clotting, immediately followed by two-step centrifugation for serum separation: centrifugation of whole blood at 5000 rpm for 10 min using a swinging bucket rotor. The supernatant was then aliquoted into 2-ml DNA LoBind microcentrifuge tubes (Eppendorf AG, Hamburg, Germany) and additionally centrifuged at 13000 rpm for 15 min at 4 °C to completely remove the cell debris and any remaining cellular content. The final supernatant was then transferred to new 2-ml LoBind tubes. Any samples with signs of haemolysis were excluded from the study. The serum samples were stored at −80 °C until further use.

## Virological assays

Viral RNA was extracted from the CHC patients’ serum samples for HCV

RNA quantification and genotyping using QIAamp viral RNA mini kit (Qiagen, Valencia, CA, USA) according to manufacturer’s instructions. The quantification was performed using an HCV RT-qPCR kit (Sacace Biotechnologies, Caserta, Italy) according to the manufacturer’s instructions on a Smart Cycler II Real-time PCR system (Cepheid, Sunnyvale, CA, USA). The HCV genotyping was performed using genotype-specific primers as reported previously[^2^](#_ENREF_2).

## Histology

Liver biopsies were independently evaluated by a pathologist, who was unaware of the study or the clinical data of the patients, according to the Ishak modified histopathology index (HAI) grading and staging system[^3^](#_ENREF_3). Necroinflammatory activity was graded on a scale of 0 to 18 (modified HAI grading) and the staging for liver fibrosis and architectural disturbances was performed using a scale of 0 to 6 (modified staging).

## Total RNA isolation

***From liver tissues:*** Total RNA including miRNA was extracted from control and CHC-positive liver tissues using a miRNeasy mini extraction kit (Qiagen, Valencia, CA, USA) following some modifications according to the tissue weight. First, the liver tissues stored in RNAlater at −80 °C were allowed to thaw at room temperature (15–20 °C) for 5 min. A fixed amount of liver tissue (3 mg) was excised from each sample using disposable sterile surgical blades and submerged into 2-ml LoBind tubes, which were pre-filled with 400 μl of QIAZOL lysis from the miRNeasy mini kit. LoBind tubes containing liver tissue samples were placed on CoolSafe aluminium chambers (Diversified Biotech, Newton Center, MA, USA) during the extraction to maintain a uniform temperature across all samples. Each sample was separately homogenized using a hand-held Tissue Master 125 tissue homogenizer (Omni International, Marietta, Georgia, USA) fixed with a sterile 5-mm stainless steel probe until no visible particles are seen. The remaining 400 μl lysis solution was then added for a final volume of 800 μl of lysis reagent, which was left at room temperature for 5 min to promote complete dissociation of nucleoprotein complexes. After this, 200 μl of chloroform was added followed by vortexing for 20 s, and incubation at room temperature for 3 min. The LoBind tubes containing homogenate were then centrifuged at 14,000 rpm at 4 °C for 15 min for phase separation. The upper transparent aqueous phase was transferred to new 2-ml LoBind tubes and 100% ethanol was added to maintain the manufacturer’s recommended 1.5 volume ratio between the collected upper aqueous phase and ethanol, and mixed thoroughly by pipetting. During the RNA binding, washing and elution steps, new collection tubes were used to avoid potential contamination and carryover of inhibitors. The miRNeasy mini spin columns were placed into 2-ml collection tubes (supplied with the kit) and 700 μl of aqueous phase plus ethanol mixture was pipetted into the spin column and centrifuged at 10,000 rpm at room temperature for 15 s. The flow-through was discarded, columns were fitted into new collection tubes and same procedure was repeated until all of the aqueous phase plus ethanol mixture was passed through the mini columns. During washing, 700 μl of buffer RWT was added to mini spin columns fixed into new collection tubes and centrifuged at 10,000 rpm at room temperature for 15 s. The second washing was performed as per the manufacturer’s instructions: 500 μl of RPE buffer was added to mini columns and centrifuged at 10,000 rpm at room temperature for 15 s. Another 500 μl of buffer RPE was then added but centrifugation was performed at 10,000 rpm at room temperature for 2 min to avoid ethanol carryover. Finally, the columns were again placed into new collection tubes and centrifuged at full speed for 2 min to ensure complete drying of the spin column membrane. To elute the total RNA including miRNAs, the spin columns were placed into 1.5-ml LoBind collection tubes. A fixed volume (35 μl) of DNAse/RNAse-free water was pipetted directly onto the middle of spin column membrane and the column was centrifuged at 10,000 rpm for 1 min at room temperature. The quality and integrity of the total RNA was assessed using NanoDrop 8000 spectrophotometer (NanoDrop technologies, Wilmington, DE, USA) and Agilent 2100 Bioanalyzer (Agilent Technologies, Santa Clara, CA, USA).

***From serum:*** Total RNA containing miRNA was extracted from the serum samples using a miRNeasy mini kit and following the manufacturer’s supplementary protocol for the isolation of miRNA from serum samples. Briefly, 200 μl of serum sample was mixed with 1000 μl QIAzol lysis reagent (1:5) in 2-ml LoBind tubes and vortexed vigorously for 5 min. The tubes containing homogenate were then placed at room temperature for 5 min for the complete dissociation of nucleoprotein complexes. In order to monitor extraction efficacy and for the minimization of sample-to-sample variation, 5ul of 5 nM of synthetic *Caenorhabditis elegans* miRNA-39 (cel-miR-39-3p) miScript mimic was added to each lysed sample. This step was followed by the addition of 200 μl of chloroform and vigorous vortexing for 1 min and placed at room temperature for 5 min. The samples were centrifuged at 12,000 rpm at 4 °C for 15 min for phase separation and the upper aqueous phase transferred to new 2-ml LoBind tubes. According to the volume of aqueous phase obtained, 1.5 volumes of 100% ethanol was added and mixed thoroughly by pipetting up and down several times. The washing steps were performed as given above for tissue samples. After the washing steps, the spin columns were placed into collection tubes and the total RNA including miRNAs was eluted in 35 μl DNAse/RNase-free water via centrifugation for 1min at 10,000 rpm and immediately stored at -80 °C until further use.

## Reverse transcription

Total RNA from tissue and serum samples was reverse transcribed using miScript II RT Kit (Qiagen, Valencia, CA, USA) following the manufacturer’s instructions. A fixed amount of 1 μg and fixed volume of 5 μl of total RNA from tissue and serum samples respectively was reverse transcribed in a final mixture of 20 μl containing 4 μl 5× miScript HiSpec Buffer, 2 μl 10× Nucleics mix, 2 μl miScript Reverse Transcriptase Mix and RNase-free water. The volume of RNase-free water was variable and adjusted according to the concentration of 1 μg of the eluted total RNA for each tissue sample. In the case of serum samples, 7 μl of RNase-free water was used. The reaction was then incubated at 37 °C for 60 min followed by 95 °C for 5 min on a thermocycler (Applied Biosystems, Foster City, CA). The synthesized undiluted cDNAs were then immediately stored in LoBind tubes at −20 °C until further use.

***Real-time quantitative PCR:*** RT-qPCR analysis for tissue and serum samples was performed using miScript SYBR Green PCR Kit (Qiagen, Valencia, CA, USA) following the manufacturer’s instructions. Custom miScript miRNA PCR arrays (Qiagen, Valencia, CA, USA) were used which were provided pre-coated with miScript Primer Assays (Qiagen, Valencia, CA, USA) for hsa-miR-122-5p (assay ID: MS00003416), cel-miR-39-3p (assay ID: assay ID: MS00019789), and RNU6B (assay ID: MS00033740). RNU6B and cel-miR-39 were used as normalization controls for miR-122 levels in tissue and serum samples respectively. As there is currently no consensus on a suitable normalization control for serum miRNA profiling, and cel-miR-39 has been shown to serve as a stable reference normalization control, we also used it as normalization control in the present study. First, the 20 μl cDNA template was diluted by adding 200 μl RNase-free water to ensure a final concentration of 1 ng per tube/well. A reaction mix of 25 μl per sample was then prepared as follows: 12.5 μl 2× QuantiTect SYBR Green PCR Master Mix, 2.5 μl 10× miScript Universal Primer, 2.5 μl diluted cDNA and 7.5 μl RNase-free water. All real-time PCR reactions were performed in duplicate on an iQ5 Cycler Multicolor real-time PCR detection system (Bio-Rad Laboratories, Hercules, CA, USA). The amplification cycling conditions were: 95 °C for 15 min, followed by 40 cycles of 94 °C for 15 s, 55 °C for 30 s and 70 °C for 30 s. The relative expression levels, or fold change, of hepatic and serum miR-122 were calculated using a 2^∆∆Cq^ method where, ∆∆Cq = ∆Cq (Cq^RNU6B^ − Cq^miR-122^)_CHC_ − ∆Cq (Cq^RNU6B^ − Cq^miR-122^)_Controls_ and ∆∆Cq = ∆Cq (Cq^cel-miR-39^ − Cq^miR-122^)_CHC_ − ∆Cq (Cq^cel-miR-39^ − Cq^miR-122^)_Controls_, respectively. The Cq values refer to the number of quantification cycles required for the fluorescent signal to cross the defined threshold level in RT-qPCR and are inversely proportional to the expression level of the miRNA in question. Therefore, the lower the Cq value, the higher the expression level of miRNAs and vice versa. For the ∆Cq values to reflect actual/direct expression instead of the opposite, a modification of the Livak method was used where the Cq values of normalizers were subtracted from that of miR-122, instead of subtracting the Cq of miR-122 from the Cq of normalization controls. All RT-qPCR reactions were performed in accordance to the Minimum Information for Publication of Quantitative Real-Time PCR Experiments (MIQE) guidelines[^4^](#_ENREF_4).

**Statistical analyses**

The differences between two or more groups were evaluated using the Mann–Whitney *U* or the Kruskal–Wallis tests, respectively. A Spearman non-parametric rank test was used to determine the correlations, computed as the correlation coefficient *r*, between the expression levels of hepatic and serum miR-122 and the clinicopathological parameters. Univariate and multivariate logistic regression analyses were performed to determine the association between hepatic and serum miR-122 levels, clinicopathological features, and with ETR. Statistical analyses were performed using SPSS software version 23 (SPSS Inc., Chicago, IL, USA). Receiver operator characteristic (ROC) curves were generated to determine the diagnostic potential of serum miR-122 via calculation of the AUC, sensitivity, and specificity ratios using MedCalc Statistical Software version 15.8 (MedCalc Software, Ostend, Belgium). *P* values were two-sided, and values less than 0.05 were considered to be statistically significant for all analyses.

**Tables**

Table 1. Demographics and clinical features of study participants

| **Parameters^a^** | **Chronic Hepatitis C Patients (Genotype 3)** | | | |  | **Reference Ranges** |
| --- | --- | --- | --- | --- | --- | --- |
| ***General features*** | **Total**  **(n= 123)** | **SVR**  **(n = 70)** | **NR^b^**  **(n = 53)** | **RR^c^**  **(n = 20)** | **Controls**  **(n= 60)** |  |
| Age (years) | 32.7 ± 9.9 (18 – 60) | 32.3 ± 8.4 (18 – 50) | 33 ± 11.3 (18 – 60) | 33.9 ± 13.9 (18 – 60) | 39.2 ± 12.9 (20 – 61) | - |
| Gender (M/F) | 61/62 | 35/35 | 26/27 | 12/08 | 30/30 |  |
| Platelet count  ( x 10^3^/µl) | 220 ± 69.4 (110 – 426) | 209.4 ± 65.9 (110 – 402) | 231.7 ± 71.1 (151 – 426) | 227.8 ± 65.9 (151 – 426) | 246.9 ± 69.5 (160 – 400) | 150 – 450 |
| ***Liver function indicators*** | | | | | |  |
| BT (mg/dl) | 0.7 ± 0.3 (0.4 – 1.6) | 0.8 ± 0.3 (0.4 – 1.5) | 0.7 ± 0.2 (0.4 – 1.6) | 0.7 ± 0.2 (0.5 – 1.1) | 0.6 ± 0.1 (0.4 – 0.9) | ≤ 1.0 |
| BD (mg/dl) | 0.4 ± 0.2 (0.1 – 0.8) | 0.4 ± 0.2 (0.1 – 0.8) | 0.3 ± 0.1 (0.2 – 0.6) | 0.3 ± 0.1 (0.2 – 0.6) | 0.2 ± 0.1 (0.1 – 0.3) | ≤ 0.3 |
| BI (mg/dl) | 0.4 ± 0.2 (0.2 – 1) | 0.4 ± 0.1 (0.2 – 0.8) | 0.4 ± 0.2 (0.2 – 1) | 0.4 ± 0.1 (0.2 – 0.6) | 0.4 ± 0.1 (0.2 – 0.7) | ≤ 0.7 |
| ALT (IU/L) | 65 ± 51.7 (13 – 397) | 73.6 ± 58.9 (13 – 397) | 55.1 ± 39.6 (13 – 187) | 71.2 ± 51 (14.5 – 187) | 22.6 ± 8.2 (8 – 40) | 10 – 40 |
| AST (IU/L) | 54.8 ± 37.5 (14 – 235) | 62.4 ± 43.3 (14 – 235) | 45.7 ± 26.3 (19.3 – 134) | 53.6 ± 35.1 (19.3 – 134) | 27.2 ± 7.4 (14 – 44) | 10 – 45 |
| AST/ALT ratio | 0.9 ± 0.3 (0.0 – 2.0) | 0.9 ± 0.3 (0.6 – 2.0) | 0.9 ± 0.3 (0.0 – 2.0) | 0.9 ± 0.3 (0.0 – 1.4) | 0.6 ± 0.1 (0.0 – 0.7) | < 1.0 |
| ALP (IU/L) | 236.5 ± 46.4 (137.7 – 325) | 220.4 ± 39.9 (137.7 – 325) | 245 ± 46 (166 – 311) | 237.5 ± 47.3 (171 – 305) | 194 ± 37.4 (90 – 240) | 80 – 290 |
| TP (g/dl) | 7.7 ± 0.4 (6.8 – 8.9) | 7.8 ± 0.4 (6.8 – 8.9) | 7.5 ± 0.4 (6.9 – 8.7) | 7.5 ± 0.4 (6.9 – 8.2) | 6.9 ± 0.3 (6.4 – 7.6) | 6.4 – 8.5 |
| Alb (g/dl) | 5.1 ± 0.4 (4.0 – 5.8) | 5.1 ± 0.3 (4.3 – 5.7) | 5 ± 0.4 (4 – 5.8) | 4.9 ± 0.4 (4 – 5.8) | 4.1 ± 0.2 (3.8 – 4.5) | 3.8 – 4.5 |
| Glb (g/dl) | 2.6 ± 0.4 (1.5 – 3.7) | 2.7 ± 0.4 (1.5 – 3.7) | 2.6 ± 0.4 (1.8 – 3.7) | 2.6 ± 0.5 (1.2 – 3) | 2.8 ± 0.3 (2.5 – 3.5) | 2.5 – 3.5 |
| A/G ratio | 2 ± 0.4 (1.2 – 3.5) | 2 ± 0.5 (1.3 – 3.5) | 2 ± 0.4 (1.2 – 3) | 2 ± 0.5 (1.2 – 3) | 1.5 ± 0.2 (1.1 – 1.7) | 1.0 – 1.8 |
| γ−GT (IU/L) | 28 ± 14.5 (7 – 91) | 25.9 ± 11.7 (7 – 55) | 30.6 ± 16.9 (7 – 91) | 31.3 ± 11.7 (7 – 53) | 23.8 ± 12.3 (6 – 44) | 5 – 45 |
| PT (seconds) | 14.8 ± 1.4 (10 – 18) | 15.2 ± 1.2 (11 – 18) | 14.4 ± 1.5 (10 – 16) | 14.7 ± 1 (13 – 16) | 14.5 ± 1.4 (11 – 16) | 11 – 16 (Control: 14) |
| APTT (seconds) | 36.9 ± 2.1 (32 – 42) | 37.1 ± 2.2 (34 – 42) | 36.5 ± 2 (32 – 41) | 35.7 ± 2.2 (32 – 40) | 37.3 ± 2.7 (33 – 43) | 32 – 45 (Control: 36) |
| INR | 1.0 ± 0.2 (0.0 – 1.3) | 1.1 – 0.2 (0.0 – 1.3) | 1 ± 0.2 (0.0 – 1.1) | 1 – 0.2 (0.0 – 1.1) | 1 – 0.1 (0.8 – 1.1) | 0.8 – 1.2 |
| ***Serum lipid measures*** | | | | | |  |
| TC (mg/dl) | 181.1 ± 39.5 (112.4 – 286.0) | 179.4 ± 43.6 (112.4 – 286) | 183 ± 34 (115 – 254) | 196.2 ± 33.9 (138 – 254) | 144.7 ± 21.8 (113 – 197) | Recommended: < 200  Moderate risk: 200 – 239  High risk: > 240 |
| TG (mg/dl) | 141.3 ± 32.7 (78 – 250) | 137.4 ± 28.2 (78 – 234) | 146 ± 36.9 (97 – 250) | 141.6 ± 31 (110 – 195) | 132.4 ± 48.6 (80 – 242) | Normal: < 250  Borderline high: 250 – 500  Hypertriglyceridemia: > 500 |
| HDL-c (mg/dl) | 38 ± 6.2 (26.6 – 56) | 36.2 ± 5.3 (26.6 – 49.7) | 40.2 ± 6.4 (27 – 56) | 43.5 ± 4.5 (33 – 52.3) | 49.4 ± 5.7 (41 – 60) | ≥ 40 |
| LDL-c (mg/dl) | 109.5 ± 29.8 (60.4 – 204) | 101.9 ± 24.8 (61.1 – 189.3) | 118.5 ± 32.7 (60.4 – 204) | 121.1 ± 34.1 (60.4 – 175.7) | 87.4 ± 22.2 (60 – 130) | ≤ 130 |
| VLDL-c (mg/dl) | 28.1 ± 6.2 (15.6 – 50) | 27.6 ± 5.3 (15.6 – 47) | 28.6 ± 7.1 (20 – 50) | 28.4 ± 5.8 (22 – 39) | 17.9 ± 6.5 (4 – 29) | ≤ 30 |
| ***Necroinflammation (Grading)*** | | | | | |  |
| Minimal  (0-3) | 46 (37.4) | 22 | 21 | 10 | - | - |
| Mild (4-8) | 66 (53.6) | 34 | 32 | 13 | - | - |
| Moderate  (9-13) | 11 (8.9) | 10 | 4 | 3 | - | - |
| Severe  (14-18) | 0 | 0 | 0 | 0 | - | - |
| ***Fibrosis (Staging)*** | | | | | |  |
| Low stage fibrosis (0-3) | 95 (77.2) | 46 | 41 | 16 | - | - |
| High stage fibrosis (4-6) | 28 (22.7) | 20 | 16 | 10 | - | - |
| ***Virus characteristics*** | | | | | |  |
| Viral load (log IU/ml) | 5.3 ± 0.9 (3.6 – 7.9) | 5.3 ± 0.9 (3.6 – 7.9) | 5.2 ± 0.9 (4.1 – 6.8) | 5 ± 1 (4.1 – 6.8) | - | - |
| ***Sugar levels profile*** | | | | | |  |
| Fasting glucose (mg/dl) | 68 ± 10 (55 – 85) | 69.5 ± 10.3 (55 – 89) | 69.8 ± 15.8 (55 – 123) | 77.1 ± 19.2 (58 – 123) | 89.4 ± 12.5 (70 – 108) | 70 - 110 |
| Fasting Insulin  (mIU/L) | 13.54 ± 3.2 (6.9 – 20.3) | 14.2 ± 3.5 (6.9 – 20.3) | 14.5 ± 2 (12.1 – 18) | 14.5 ± 1.7 (12.1 – 16) | 8.6 ± 1.3 (7 – 11) | 3 - 28 |
| HOMA-IR | 2.4 ± 0.5 (1.3 – 3.3) | 2.4 ± 0.5 (1.3 – 3.7) | 2.5 ± 0.6 (1.7 – 4.2) | 2.7 ± 0.6 (2.2 – 4.2) | 1.9 ± 0.1 (1.7 – 2.0) | 1.7 – 2.0 |

Clinical parameters are given as mean ± SD (range) of no.(%) of patients. ^a^clinical parameters; ^b^complete non-responders including relapsers; ^c^relapsers only excluding non-responders; M, male; F, female; BT, bilirubin total; BD, bilirubin direct; BI, bilirubin indirect; ALT, alanine aminotransferase; AST, aspartate aminotransferase; ALP, alkaline phosphatase; TP, total proteins; Alb, albumin; Glb, globulin; γ-GT, gamma-glutamyl transferase; PT, prothrombin time; APTT, activated partial thromboplastin time; INR, international normalized ratio; TC, total cholesterol; TG, triglycerides; HDL-c, high density lipoprotein cholesterol; LDL-c, low density lipoprotein cholesterol; VLDL-c, very low density lipoprotein cholesterol; SVR, sustained virological responder; NR, non-responder; RR, relapser.

**References**

1 European Association for the Study of the, L. EASL Clinical Practice Guidelines: management of hepatitis C virus infection. *J Hepatol*. **55**, 245-264 (2011).

2 Idrees, M. Development of an improved genotyping assay for the detection of hepatitis C virus genotypes and subtypes in Pakistan. *J Virol Methods*. **150**, 50-56 (2008).

3 Ishak, K. *et al.* Histological grading and staging of chronic hepatitis. *J Hepatol*. **22**, 696-699 (1995).

4 Bustin, S. A. *et al.* The MIQE guidelines: minimum information for publication of quantitative real-time PCR experiments. *Clin Chem*. **55**, 611-622 (2009).
